# The Self-Administered Gerocognitive Examination (SAGE): Equivalence of Parallel Versions and Validity in Cognitively Unimpaired Controls and Patients With Mild Cognitive Impairment or Dementia in a Memory Clinic

**DOI:** 10.1097/WAD.0000000000000673

**Published:** 2025-05-15

**Authors:** Roy P.C. Kessels, Floor S. van Bergen, Iris J. Harmsen, Daan K.L. Sleutjes, Paul L.J. Dautzenberg, Joukje M. Oosterman

**Affiliations:** * Radboud University, Donders Institute for Brain, Cognition and Behaviour, Nijmegen; † Radboud University Medical Center, Radboudumc Alzheimer Center, Nijmegen; ‡ Vincent van Gogh Institute for Psychiatry, Venray; § Jeroen Bosch Hospital, Department of Medical Psychology; ‖ Jellinek, Amsterdam; ¶ Jeroen Bosch Hospital, Department of Geriatric Medicine, ’s-Hertogenbosch; # Brain Research Center, ’s-Hertogenbosch

**Keywords:** MCI, dementia, cognitive screening, neuropsychology, primary-care assessment

## Abstract

**Objective:**

Cognitive screens to diagnose mild cognitive impairment (MCI) or dementia require supervision and cannot be easily administered in primary care. Here, we validated the Self-Administered Gerontocognitive Examination (SAGE), investigating the alternate version equivalence, the convergent validity using neuropsychological tests, and its diagnostic accuracy.

**Patients:**

Thirty-two MCI patients and 34 with dementia were recruited from a memory clinic in the Netherlands, and 69 healthy controls over the age of 50.

**Methods:**

The 4 alternate versions of the SAGE were compared. Receiver operating characteristic (ROC) analyses were performed, comparing the controls to the MCI and dementia groups. Associations between SAGE scores and standard neuropsychological tests were examined.

**Results:**

No performance differences were found between the alternate versions. Performance differences were found on the SAGE between the 3 groups, with fair to good areas under the curve. A cutoff score of <18 had the best diagnostic accuracy for controls versus dementia, <20 for controls versus MCI and <19 for controls versus cognitively impaired. SAGE scores correlated with standard neuropsychological tests.

**Discussion:**

The SAGE is a valid tool for distinguishing cognitively unimpaired individuals from people with dementia or MCI.

Early detection of mild cognitive impairment (MCI) or dementia is important, as a timely diagnosis may help to optimize patient care and provide adequate support for the patient and caregiver. Also, a formal diagnosis may give a patient access to new potentially disease-modifying drugs that are currently becoming available. However, not all individuals with dementia receive a formal diagnosis; a recent systematic review found a pooled prevalence of undetected dementia of 61.7%. Possible reasons for this underdiagnosis include low socioeconomic status (including low income), a low education level, living in a rural area, poor access to health care, limited capacity of specialized memory clinics, national health policies, and cultural factors.^1^ This illustrates the need for easy-to-use and accessible sensitive and specific screening tools that can be applied to identify individuals with suspected MCI or dementia.

Traditional cognitive screens used to identify such individuals include the Mini-Mental State Examination (MMSE) and Montreal Cognitive Assessment (MoCA). While such instruments have been demonstrated to be valid in detecting dementia, they all have important shortcomings.^2^ For example, the MMSE is not sensitive to subtle cognitive decline (as is the case in MCI), is biased towards the Alzheimer-type cognitive dysfunction (not including measures of executive function and speed of processing) and does not have alternate forms for repeated assessments. The MoCA is considered to have superior psychometric characteristics compared with the MMSE, including 3 parallel versions for repeated assessment, especially for the detection of MCI, but performance on the MoCA is still very much influenced by age and (low) educational attainment.^3^ In addition, these cognitive screens all have to be administered by a trained health care professional. Especially in primary health care, resources are limited and specialized staff is not always available to administer such tests.

An instrument to overcome these limitations and that is feasible for use in primary care settings is the Self-Administered Gerocognitive Examination (SAGE; https://wexnermedical.osu.edu). The SAGE is a self-administered paper-and-pencil instrument that assesses cognitive functioning by testing 6 cognitive domains (language, reasoning/computation, visuospatial abilities, executive functioning, memory, and orientation). The SAGE comes in 4 equivalent alternate forms to prevent material-specific test-retest effects. Previous research^4^ has shown that the original version of the SAGE has excellent sensitivity (95%) and moderate specificity (62%) in differentiating between 21 healthy participants and 21 patients with MCI, and a high sensitivity and specificity (both 95%) in discriminating 21 controls from 21 patients with dementia, with a cutoff score of ≤16. The SAGE was found to be superior to the MMSE in detecting conversion from MCI to dementia.^5^ Application in primary care settings resulted in a 15-fold increase in detecting cognitive conditions compared with informant-based reports.^6^ However, substantial age and education effects have been reported in a large community sample (n=1047) over 50 years of age, with 28.4% identified as having cognitive impairment.^7^ Even though this may reflect the typical prevalence of cognitive impairment,^8^ it may also indicate the poor specificity already outlined in the original study.^4^ Moreover, although the SAGE has since been translated into multiple languages, validation studies in MCI and dementia patients so far have only been conducted by the original developers of the English-language SAGE.

The aim of the present study is therefore to further validate the SAGE by: (1) examining age and education effects and the equivalence of the 4 alternate versions in a sample of healthy individuals using the Dutch-language version of the SAGE; (2) establishing its sensitivity and specificity in a larger sample of healthy individuals, patients with MCI and patients with dementia; (3) demonstrating the convergent and divergent validity by comparing SAGE total and subscores against standard neuropsychological tests in MCI and dementia patients.

## METHODS

### Participants

A total of 135 individuals enrolled in this study, 32 of whom were patients with a diagnosis of single or multidomain MCI, 34 were patients with an all-cause dementia diagnosis, and 69 were cognitively unimpaired individuals. All participants were 50 years of age or older and had sufficient mastery of the Dutch language. Patients were visiting the geriatric outpatient clinic of the Jeroen Bosch Hospital for a neuropsychological examination. Patients were excluded from the study if they had limitations in vision, primary anxiety or mood disorders, alcohol and/or drug abuse, or severe fatigue. The participants without a diagnosis of MCI or dementia (cognitively unimpaired controls) were recruited from the waiting room of the same outpatient clinic. Control participants were included if they had no subjective cognitive complaints and were living independently at home, and were excluded if they had a psychiatric or neurological disorder or were using psychotropic drugs.

Clinical diagnoses were based on a multidisciplinary approach and used the results of a clinical interview, neuropsychological assessment, medical examination, and, if available, additional examinations such as neuroimaging. Diagnoses were made in accordance with the standard clinical criteria for MCI (ie, cognitive impairments in one or more domains, without everyday functional limitations, not caused by psychiatric disorders or delirium)^8^ and all-cause dementia (ie, cognitive impairments in one or more domains with evidence for functional decline in everyday life, not caused by psychiatric disorders or delirium).^9^ Education level was categorized for all participants as low, average, or high in accordance with the Dutch educational system.^10^ The estimated years of education for comparison with the Anglo-Saxon educational system are presented for descriptive purposes.

### SAGE Test Description

The SAGE is a cognitive screen that measures the level of cognitive functioning. It consists of 6 domains: language (picture naming; verbal fluency), reasoning/computation (abstraction; calculations), visuospatial abilities (copying 3-dimentional constructions; clock drawing), executive functioning (modified Trail Making Test B; mental flexibility), memory, and orientation (date). The total test adds up to 12 questions with a maximum score of 22 points. Detailed scoring instructions are available in Dutch. It is a self-administered tool; no training is required to complete the test and assistance is prohibited. Patients are required to have sufficient vision and written language skills to take the test (though correct spelling is not required). In addition, the SAGE has 4 alternate forms (forms 1 through 4) that have minor differences in the questions regarding fluency, picture naming, construction, memory, abstraction, calculation, and problem solving, to overcome material-specific practice effects.

### Neuropsychological Assessment

The patients visited the geriatric outpatient clinic for an extensive neuropsychological examination, including a set of neuropsychological tests as part of clinical routine care. The tests were adjusted slightly per patient, depending on suspected diagnosis (ie, in case of suspected memory impairments, an extra memory test might be added). A minimal data set of 5 validated and reliable neuropsychological tests was selected for comparison with the Dutch SAGE, which were administered to most patients. These were selected to cover several domains and based on the CERAD:^11^ the Rey Auditory Verbal Learning Test (RAVLT; total immediate and delayed recall score) for episodic memory,^12^ Concept Shifting Test (CST) part C for concept shifting and executive functioning,^13^ the Letter Digit Substitution Test (LDST) for psychomotor speed,^14^ the Boston Naming Test (BNT) short form^15^ for language (naming), and verbal fluency (both animal and profession categories) for executive functioning and language.^10^ Furthermore, the MMSE was available for a subsample of the patients (as this was not part of the neuropsychological assessment, but administered during another visit that was 1 month or less apart from the neuropsychological assessment).

### Procedure

The 4 alternate versions of the SAGE were administered in the cognitively unimpaired participants, allocated in a pseudo-random version. Version 1 was administered in 18 controls, the other 3 versions in 17 controls each. All patients completed version 2 of the SAGE because of item overlap between SAGE versions 1, 3, and 4 and the standard neuropsychological tests. Table 1 shows the participant characteristics for the 3 groups.

**TABLE 1:** Participant Characteristics for the Patients With MCI or Dementia and the Cognitively Unimpaired Controls

	MCI (*n*=32)	Dementia (*n*=34)	Controls (*n*=69)
Age*	71.5 (6.5)‡	77.3 (6.4)§ ∥	64.8 (10.5)
Sex distribution (m:f)†	17:15‡	19:15	23:46
Education (low:average:high)†	14:6:9‡	18:10:5§	13:25:31
Years of education equivalent*	10.0 (4.0)	8.4 (3.3)§	11.1 (3.3)
Dementia type (n)			
Alzheimer dementia		18	
Vascular/subcortical dementia		9	
Mixed Alzheimer/vascular dementia		4	
Unknown etiology		3	

* Note. Mean (SD);

† Frequency (n);

‡ *P* < 0.05,

§ *P* < 0.01 compared with controls;

∥ *P* < 0.01 compared with MCI.

### Statistical Analyzes

IBM SPSS 29.0.0 was used for all analyzes (α=0.05). Nonparametric analyzes were performed as the SAGE total score and subscores are not normally distributed. First, the equivalence of the alternate forms of the SAGE was examined using the Kruskal-Wallis *H* test. Next, a multiple linear regression was used to examine the effect of age, education, and sex on the SAGE total score in the healthy controls. Overall, between-group differences for the SAGE total score and the subdomain scores were analyzed using the Kruskal-Wallis *H* test, followed by comparisons between individual groups using Mann-Whitney *U* tests. Subsequently, receiver operating characteristics (ROC) curves were determined comparing controls versus MCI, controls versus dementia, and controls versus cognitively impaired (CI), that is, MCI and dementia taken together. Sensitivity and specificity values for different cutoff points were obtained, and positive (PPV) and negative predictive values (NPV) were computed for a prevalence (base rate) of 10%, 25%, and 50%.^16^ Area under the curve (AUC) values between 0.6 and 0.7 are often interpreted as poor, 0.7 and 0.8 as fair, between 0.8 and 0.9 as good, and between 0.9 and 1.0 as excellent.^17^ The optimal cutoff point was identified using Youden's *J* statistic,^18^ where the maximum score was obtained with the following formula: *J*=sensitivity+specificity –1 (*J*>0.5 is considered acceptable diagnostic accuracy). Finally, Spearman ρ rank correlations were computed between the SAGE (total score and domain scores) and the selected neuropsychological tests. ρ=0.5 is seen as large, ρ=0.3 as medium, and ρ=0.1 as small.^19^

## RESULTS

Table 1 shows the demographic variables of the 3 groups. Age differed across the groups [*F*(2,132) = 24.2, *P*<.001]. The controls were younger than the MCI (*P*<.001) and dementia (*P*=.001) groups, and the MCI group was younger than the dementia group (*P*=.008). Also, the sex distribution of the groups differed [χ^2^(2)=6.22, *P*=.045]. Specifically, the control group consisted of significantly more women than the dementia group [χ^2^(1)=4.80, *P*=0.029]. There were also differences in the education level of the groups [*H*(2) = 15.4, *P*<.001], that is, the control group consisted of more people with a high education level compared with the MCI (*U*=725.5, Z=2.28, *P*=.022) and dementia (*U*=639.5, *Z*=3.79, *P*<.001) group, and the dementia group had less years of education than the controls (*P*<.001).

**FIGURE 1:**
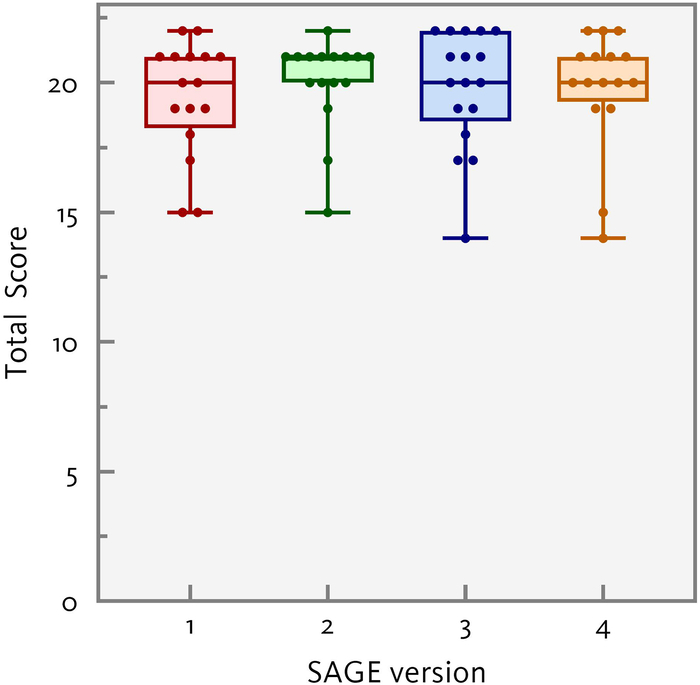
Box plots showing the score distributions for the 4 parallel version of the SAGE; the hinges of the box represent the 25th and 75th percentile, the line in the middle of the box represents the median, and the whiskers show the minimum and maximum values.

Figure 1 shows the box and whisker plots for the 4 parallel versions of the SAGE for the control group. No significant differences were found in the score distributions across the 4 alternate versions [*H*(3)=1.53, *P*=0.68], indicating that there are no systematic differences between the 4 versions. As a result, the SAGE scores for the complete control group were taken together in all subsequent analyzes. The linear regression model with age, education level and sex as predictors for the SAGE total score was significant [*F*(3,65)=14.3, *P*<.001, *R* ^2^=0.398]. Age (*B*=−.11, *P*<.001, 95% CI: −.154 to −.057) and education level (*B*=1.47, *P*<.001, 95% CI: .777-2.168) were significant predictors, while sex was not (*B*=−.05, *P*=.623, 95% CI: −1.399 to .844).

Table 2 shows the results for the SAGE total score and the 6 domain scores for the MCI, dementia and control groups. Overall group differences were found for the SAGE total score and all domain scores (*H*-values >13.0, *P*-values<.002). MCI patients performed worse than controls on the SAGE total score (*U*=366.5, *Z*=5.43, *P*<.001), as well as the orientation (*U*=946.5, *Z*=1.97, *P*<.05), language (*U*=777.5, *Z*=3.22, *P*=.001), memory (*U*=663.5, *Z*=3.55, *P*<.001), reasoning (*U*=841.0, *Z*=2.32, *P*<.05), and executive (*U*=468.5, *Z*=4.90, *P*<.001) domains. The dementia group performed worse than the controls on the SAGE total score and all subdomains (*U*-values >121.5, *Z*-values >4.89, *P*-values <.001). The dementia patients also performed worse on the SAGE total score and all domain scores than the MCI group (*U*-values >207.0, *Z*-values >2.01, *P*-values <.05), apart from Memory (*U*=470.5, *Z*=1.12).

**TABLE 2:** Means and SD for the SAGE Total Score and Domain Scores for the MCI, Dementia, and Control Groups

	Mean (SD*)*		
SAGE score	MCI (n=32)	Dementia (n*=*34)	Controls (n=69)
Total score	15.9† (2.9)	11.5† § (3.9)	19.4 (2.6)
Orientation	3.6* (0.8)	3.2† ‡ (0.8)	3.9 (0.4)
Language	3.5† (0.7)	2.3† § (1.2)	3.8 (0.4)
Memory	0.7† (0.9)	0.4† (0.8)	1.4 (0.8)
Reasoning	3.1* (1.1)	2.0† § (1.2)	3.7 (0.6)
Executive	1.9† (1.2)	1.2† ‡ (1.3)	3.3 (1.1)
Visuospatial	3.1 (1.0)	2.4† ‡ (1.4)	3.4 (0.9)

* *P*<0.05.

† *P*≤0.01 compared with controls.

‡ *P*<0.05.

§ *P*≤0.01 compared with MCI (Mann-Whitney *U* test).

**FIGURE 2:**
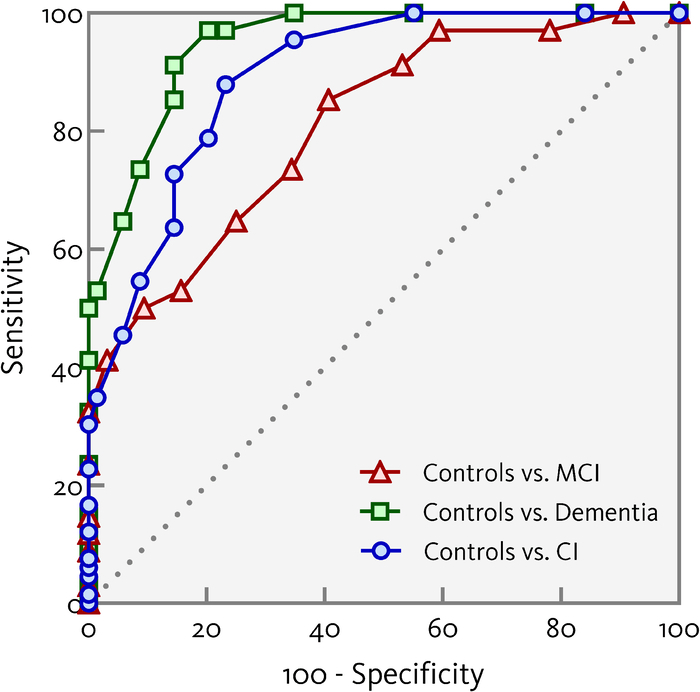
Receiver operating characteristics curves showing the sensitivity and specificity for controls versus the mild cognitive impairment (MCI) and dementia groups, and for controls versus cognitively impaired (CI; taking the MCI and dementia groups together), using the SAGE total score (uncorrected for age or education level).

Figure 2 shows the ROC curves for the SAGE total score. Comparing the control versus the dementia group resulted in an AUC of .955 (*P*<.0005; 95% CI: .992-.989) and an optimal cutoff score of <18 (sensitivity=1.000, specificity=.797, *J*=.797). Comparing the control versus the MCI group showed an AUC of .834 (*P*<.0005, 95% CI: .758-.910). Here, a cutoff <20 resulted in the highest Youden index (*J*=.558), with a sensitivity of .906, and a specificity of .652. Comparing the control versus the CI group resulted in an AUC of .896 (*P*<.0005, 95% CI: .844-947), with an optimal cutoff of <19 (sensitivity .892, specificity .768, *J*=.660). The ROC analyzes were also performed with an age and education correction proposed previously,^7^ by adding 1 point to the SAGE total score for individuals older than 80 and adding 1 point for individuals with 12 years or less education. These analyzes did not alter the test’s diagnostic overall accuracy (see Supplementary file 1) Appendix A, Supplemental Digital Content 1, http://links.lww.com/WAD/A523 shows the sensitivity and specificity of the best cutoff scores for each comparison, Appendix B, Supplemental Digital Content 1, http://links.lww.com/WAD/A523 shows the corresponding PPV and NPV.

Correlational analyzes between the SAGE and the neuropsychological test showed that the SAGE total score correlated strongly with the MMSE and moderately with the CST-C, BNT, and animal fluency. SAGE Language correlated strongly with the MMSE and moderately with the BNT and animal fluency. The SAGE Memory domain correlated moderately with the MMSE. SAGE executive correlated moderately with the CST-C and animal fluency, and SAGE visuospatial was moderately associated with the MMSE, CST-C, and LDST (Table 3).

**TABLE 3:** Spearman Correlations Between SAGE Total Score and Subscores and Neuropsychological Tests, Available in a Subset of the Patients With MCI or Dementia

		RAVLT (*n* = 64)					Verbal fluency (*n* = 66)	
SAGE score	MMSE (*n* = 29)	Immediate recall	Delayed recall	CST-C (*n* = 58)	LDST (*n* = 40)	BNT-sf (*n* = 61)	Animal	Profession
Total score	0.80†	0.01	0.02	0.38†	0.26	0.30*	0.35†	0.03
Language	0.62†	−0.10	0.06	0.23	0.18	0.33*	0.32†	0.11
Memory	0.48†	0.00	0.12	0.04	−0.13	0.19	0.06	0.08
Executive	0.30	0.07	−0.02	0.44†	0.28	0.04	0.31*	0.11
Visuospatial	0.40*	−0.08	−0.07	0.40†	0.38*	0.01	0.20	−0.17

* *P* < 0.05,

† *P* < 0.01.

BNT-sf indicates Boston naming test-short form; CST-C, concept shifting task part C; LDST, letter digit substitution test; MMSE, mini-mental state examination; RAVLT, Rey auditory verbal learning test; SAGE, self-administered gerocognitive examination.

## DISCUSSION

In this study, we validated the SAGE in a sample of patients with MCI or dementia diagnosed in a memory clinic in the Netherlands. We also examined whether significant differences were present between the 4 alternate forms of the SAGE using a sample of cognitively healthy volunteers over the age of 50, also examining age and education effects in these controls. We first demonstrated that there were no differences in performance between the 4 alternate versions of the SAGE, suggesting that these are indeed equivalent. Using alternate forms of cognitive tests is highly recommended for repeated assessments, as material-specific practice effects may occur, especially for tests that measure word memory or verbal fluency.^20^ We also showed that age and education affected the performance on the SAGE. This replicates a previous study that examined age and education effects in a large sample of healthy individuals in the United States.^7^ Age and education effects are strong predictors of cognitive performance on any test, and such effects have also been reported for other cognitive screens.^3^

With respect to the diagnostic accuracy, our findings show that the SAGE total score is able to distinguish cognitively unimpaired controls from people with MCI and dementia at a group level, with good to excellent AUCs. At the individual level, a cutoff SAGE total score of <18 had an excellent sensitivity and specificity in distinguishing cognitively unimpaired older adults or from dementia patients, a cutoff score of <20 for distinguishing controls from MCI patients (albeit with a low specificity of 55.8%), and a cutoff score <19 for controls versus MCI+dementia patients taken together. Applying age and education correction^7^ did not improve the SAGE’s diagnostic accuracy. Our results are in line with findings reported in the literature on other cognitive screens, such as the MMSE or MoCA, which typically show that cognitive screens have a higher diagnostic accuracy for dementia than for classifying MCI.^21–23^ Here, the specificity of possible cutoff scores is usually insufficient, especially in individuals with lower education levels. Our findings are also similar with those of Scharre et al^4^ who validated the SAGE in a smaller sample of MCI patients, dementia patients and unimpaired controls. They found a score of <17 to represent the best diagnostic accuracy for discriminating between controls and dementia patients, but also reported a low specificity (62%) for discriminating controls from MCI patients. Furthermore, our results show that even for the optimal cutoff scores, the PPV is low, especially for the 10% prevalence (which is likely the most accurate base rate for the target primary-care setting). However, for an instrument to detect cognitive impairment in primary care, a high NPV is more important than a high PPV.^24^ Overall, we found a high NPV for the different cutoff scores for all base rates comparing controls versus dementia or CI. Also, the PPV substantially increases for the high prevalences (25% to 50%) that are more in line with the higher base rate expected in a memory clinic.

Examining the SAGE total score and its subdomains, we found that the SAGE total score differed across the 3 diagnostic groups, but that the subdomain score for visuospatial function did not differ between the MCI and control groups. This illustrates that sum scores of tests are psychometrically more reliable than subscores, which often also have a very limited scoring range. Consequently, drawing conclusions about cognitive profiles derived from screeners lacks validity and should be avoided in clinical practice. Still, at a group level, the cognitive domains that are commonly affected in MCI patients (ie, problems in memory and in executive function and, closely related to the latter, abstract reasoning)^25^ showed differences between MCI patients and controls in the SAGE, adding to its validity. Furthermore, correlations between the SAGE total and subdomain scores with established neuropsychological tests emphasized the convergent validity, with strong correlations between the SAGE and the MMSE, but also significant correlations between the SAGE Language with BNT and animal fluency, SAGE executive with measures of concept shifting and animal fluency, and SAGE visuospatial with tests with a strong visuospatial processing component (ie, CST-C and LDST). Interestingly, SAGE memory did not correlate with an established word-list learning task (RAVLT); this is likely the result of the nature of the SAGE memory subtest, which essentially is a prospective memory task (ie, the instruction midway the test to memorize the instruction to write “I am done” at the bottom of the very last page of the test after finishing it). Prospective memory tests (assessing to ability to recall a planned intention and to act in accordance in a future point in time) differ from traditional episodic memory tests requiring individuals to encode and later remember words, pictures, or stories. Furthermore, the scoring range of the SAGE memory test is highly restricted, with only 0, 1, or 2 points that can be scored. However, self-assessment of memory function is notably challenging, as it requires either assistance from others to present to be remembered stimuli^26^ or relies on online computerized test paradigms.^27^

Our study also has some limitations. First, we included patients based on the syndromal classifications MCI and dementia, based on the established criteria for single or multidomain MCI^8^ and all-cause dementia.^9^ We thus do not have biomarker information to support the underlying etiology, adopting a similar design as Scharre et al^4^ Still, it should be stressed that cognitive screens are poor at distinguishing different types of dementia. Furthermore, distinguishing MCI or dementia subtypes would also require much larger samples. Also, our diagnostic groups differed with respect to age and educational attainment, but taking the proposed correction for age and education into account did not alter our findings. Second, neuropsychological test data were only available from a subsample of the patient sample, as these were collected as part of routine assessment. However, ours is the first study to correlate SAGE subdomain scores to the performance on established neuropsychological tests, showing its convergent validity. Finally, our patient sample was recruited from a single memory clinic in the Netherlands, limiting the external validity. However, the American version of the SAGE has already been validated in a community sample^7^ and in primary care settings,^6^ with promising findings.

In sum, we showed that the SAGE is a valid and easy-to-use self-administered tool for identifying people with CI in a memory clinic in the Netherlands, but that interpretation of test results for individuals suspected of having subtle cognitive impairment (ie, MCI) should be done with caution. It should be stressed that short cognitive screens can never replace more extensive neuropsychological assessments, and that a diagnosis of MCI or dementia should not solely be based on a single test outcome. The course of the symptoms, contextual factors such as age, education, and socioeconomic status, and—more importantly—functional status (ie, assessment of everyday activities) are key factors that should be considered when interpreting the outcome of a cognitive screen such as the SAGE. Related to this, it would be interesting to compare the SAGE to other tools that have been developed for use in primary care, such as the informant-based Symptoms of Dementia Screener^28^ or BASIC-Q.^16^ Also, future research could also compare the SAGE to other self-assessment tools, such as the computerized Thoven Cognitive Self-Assessment^29^ or the MoCA XpressO.^30^ Finally, normative data that allow for a more fine-grained adjustment for age and education should be collected.

## Supplementary Material
