# Supplementary material for: The Self-Administered Gerocognitive Examination (SAGE): Equivalence of Parallel Versions and Validity in Cognitively Unimpaired Controls and Patients With Mild Cognitive Impairment or Dementia in a Memory Clinic
Source: Alzheimer Dis Assoc Disord. 2025 May 15;39(2):87–92. doi: 10.1097/WAD.0000000000000673 (PMC12101880; doi:10.1097/WAD.0000000000000673)
Supplement: Supplementary file 1 [file wad-39-87-s001.docx]

**Appendix A.** The best cut off-scores for the SAGE total score (uncorrected for age or education level) for distinguishing controls from patients with mild cognitive impairment (MCI) and dementia patients, and controls from cognitively impaired patients (CI; i.e. taking MCI and dementia together). In bold the optimal values based on the Youden index.

|  |  | Controls vs. MCI | | |  | Controls vs. dementia | | |  | Controls vs. CI | | |
| --- | --- | --- | --- | --- | --- | --- | --- | --- | --- | --- | --- | --- |
| SAGE cut-off |  | Sensitivity | Specificity | Youden *J* |  | Sensitivity | Specificity | Youden *J* |  | Sensitivity | Specificity | Youden *J* |
| < 20 |  | **.906** | **.652** | **.558** |  | 1.00 | .652 | .652 |  | .954 | 0.652 | .606 |
| < 19 |  | .781 | .768 | .549 |  | 1.00 | .768 | .768 |  | **.892** | **0.768** | **.660** |
| < 18 |  | .594 | .797 | .391 |  | **1.00** | **.797** | **.797** |  | .800 | 0.797 | .597 |
| < 17 |  | .531 | .855 | .386 |  | .939 | .855 | .794 |  | .738 | 0.855 | .594 |
| < 16 |  | .406 | .855 | .261 |  | .879 | .855 | .734 |  | .646 | 0.855 | .501 |
| < 15 |  | .344 | .913 | .257 |  | .758 | .913 | .671 |  | .554 | 0.913 | .467 |
| < 14 |  | .250 | .942 | .192 |  | .667 | .942 | .609 |  | .462 | 0.942 | .404 |

**Appendix B.** Positive (PPV) and negative predictive values (NPV) for the different cut-off scores of the SAGE total score (uncorrected for age and education) for a prevalence (base rate) of 10%, 25% and 50%.

|  |  | **Prevalence 10%** | | | | | | | |
| --- | --- | --- | --- | --- | --- | --- | --- | --- | --- |
|  |  | Controls vs. MCI | |  | Controls vs. dementia | |  | Controls vs. CI | |
| SAGE cut-off |  | PPV | NPV |  | PPV | NPV |  | PPV | NPV |
| < 20 |  | **.225** | **.984** |  | .242 | 1.000 |  | .234 | .992 |
| < 19 |  | .272 | .969 |  | .317 | .996 |  | **.296** | **.983** |
| < 18 |  | .245 | .946 |  | **.347** | **.996** |  | .301 | .971 |
| < 17 |  | .289 | .943 |  | .411 | .989 |  | .358 | .966 |
| < 16 |  | .238 | .928 |  | .395 | .981 |  | .328 | .955 |
| < 15 |  | .305 | .151 |  | .484 | .969 |  | .411 | .948 |
| < 14 |  | .324 | .919 |  | .554 | .960 |  | .466 | .940 |
|  |  | **Prevalence 25%** | | | | | | | |
| < 20 |  | **.465** | **.954** |  | .489 | 1.000 |  | .478 | .977 |
| < 19 |  | .529 | .913 |  | .583 | .987 |  | **.558** | **.950** |
| < 18 |  | .494 | .855 |  | **.615** | **.988** |  | .564 | .919 |
| < 17 |  | .550 | .846 |  | .677 | .967 |  | .626 | .904 |
| < 16 |  | .483 | .812 |  | .662 | .946 |  | .594 | .876 |
| < 15 |  | .569 | .807 |  | .738 | .912 |  | .677 | .858 |
| < 14 |  | .590 | .790 |  | .788 | .889 |  | .723 | .838 |
|  |  | **Prevalence 50%** | | | | | | | |
| < 20 |  | **.723** | **.874** |  | .742 | .799 |  | .733 | .935 |
| < 19 |  | .771 | .778 |  | .807 | .963 |  | **.791** | **.864** |
| < 18 |  | .745 | .662 |  | **.827** | **.964** |  | .795 | .790 |
| < 17 |  | .786 | .646 |  | .863 | .907 |  | .834 | .758 |
| < 16 |  | .737 | .590 |  | .855 | .853 |  | .815 | .702 |
| < 15 |  | .798 | .582 |  | .894 | .775 |  | .863 | .668 |
| < 14 |  | .557 | .596 |  | .918 | .728 |  | .887 | .633 |
